# The LQB-223 Compound Modulates Antiapoptotic Proteins and Impairs Breast Cancer Cell Growth and Migration

**DOI:** 10.3390/ijms20205063

**Published:** 2019-10-12

**Authors:** Lauana Greicy Tonon Lemos, Gabriel Mello da Cunha Longo, Bruna dos Santos Mendonça, Marcela Cristina Robaina, Mariana Concentino Menezes Brum, Caíque de Assis Cirilo, Etel Rodrigues Pereira Gimba, Paulo Roberto Ribeiro Costa, Camilla Djenne Buarque, Gabriela Nestal de Moraes, Raquel Ciuvalschi Maia

**Affiliations:** 1 Laboratório de Hemato-Oncologia Celular e Molecular, Programa de Hemato-Oncologia Molecular, Instituto Nacional do Câncer (INCA). Praça da Cruz Vermelha, 23, 6 andar, Rio de Janeiro (RJ) 20230 130, Brazil; lauanalemos@gmail.com (L.G.T.L.); cunhalongo@live.com (G.M.d.C.L.); bsmendonca88@gmail.com (B.d.S.M.); mrobaina@ymail.com (M.C.R.); caiqueassis11@gmail.com (C.d.A.C.); gabinestal@yahoo.com.br (G.N.d.M.); 2 Programa de Pós-Graduação Strictu Sensu em Oncologia, INCA. Rua André Cavalcanti, 37, 2° andar, Centro, RJ 20 231-050, Brazil; mariana.concentino@gmail.com; 3 Programa de Oncobiologia Celular e Molecular, INCA. Praça da Cruz Vermelha, 23, 6 andar, Centro, RJ 20 231-050, Brazil or; 4 Departamento de Ciências da Natureza, Instituto de Humanidades e Saúde, Universidade Federal Fluminense (UFF), Rua Recife 1-7, Bela Vista, Rio das Ostras, RJ 28880-000, Brazil; 5 Laboratório de Química Bioorgânica, Instituto de Pesquisas de Produtos Naturais (IPPN), Universidade Federal do Rio de Janeiro, CCS, Bloco H - Ilha do Fundão, RJ 21941-902, Brazil; prrcosta2011@gmail.com; 6 Departamento de Química, Pontifícia Universidade Católica do Rio de Janeiro, Rua Marquês de São Vicente 225, Gávea, RJ 22435-900, Brazil; camilla-buarque@puc-rio.br

**Keywords:** breast cancer, drug resistance, LQB-223 compound

## Abstract

Drug resistance represents a major issue in treating breast cancer, despite the identification of novel therapeutic strategies, biomarkers, and subgroups. We have previously identified the LQB-223, 11a-N-Tosyl-5-deoxi-pterocarpan, as a promising compound in sensitizing doxorubicin-resistant breast cancer cells, with little toxicity to non-neoplastic cells. Here, we investigated the mechanisms underlying LQB-223 antitumor effects in 2D and 3D models of breast cancer. MCF-7 and MDA-MB-231 cells had migration and motility profile assessed by wound-healing and phagokinetic track motility assays, respectively. Cytotoxicity in 3D conformation was evaluated by measuring spheroid size and performing acid phosphatase and gelatin migration assays. Protein expression was analyzed by immunoblotting. Our results show that LQB-223, but not doxorubicin treatment, suppressed the migratory and motility capacity of breast cancer cells. In 3D conformation, LQB-223 remarkably decreased cell viability, as well as reduced 3D culture size and migration. Mechanistically, LQB-223-mediated anticancer effects involved decreased proteins levels of XIAP, c-IAP1, and Mcl-1 chemoresistance-related proteins, but not survivin. Survivin knockdown partially potentiated LQB-223-induced cytotoxicity. Additionally, cell treatment with LQB-223 resulted in changes in the mRNA levels of epithelial-mesenchymal transition markers, suggesting that it might modulate cell plasticity. Our data demonstrate that LQB-223 impairs 3D culture growth and migration in 2D and 3D models of breast cancer exhibiting different phenotypes.

## 1. Introduction

Breast neoplasia is the leading cause of deaths by cancer, in women worldwide [1]. Breast tumors are highly heterogeneous, presenting distinct clinical outcomes between molecular subgroups [2,3]. The understanding of the biology of this disease contributed to the design of novel therapeutic approaches, identification of biomarkers, and better patient stratification [4]. Anthracycline-based chemotherapeutic protocols, such as doxorubicin (DOX), have been the standard care for early and advanced breast cancer for decades [5]. Despite being one of the most potent antineoplastic drugs, chemoresistance eventually arises, which represents a major hurdle in treating breast cancer [6]. Moreover, patients receiving DOX present severe side effects, such as cardiotoxicity, which is an emerging issue, considering the increasing population of cancer survivors [7,8]. Therefore, we urge the identification of novel anticancer compounds which might be able to surpass drug resistance mechanisms in breast cancer, with tolerable associated toxicity to normal tissues. In this context, the compound 11a-N-Tosyl-5-deoxi-pterocarpan, LQB-223, has been previously demonstrated to sensitize P-glycoprotein-overexpressing multidrug-resistant tumors, with little cytotoxicity to normal cells [9,10]. We have also shown that LQB-223 presents both cytostatic and cytotoxic antitumor effects in breast cancer cell lines resembling distinct molecular subtypes in the clinical setting and irrespective of TP53 status [10]. LQB-223 antitumor activity involved suppression of proliferation, G2/M arrest, and apoptosis induction, which suggests that it might exhibit pleiotropic effects towards breast cancer cells. LQB-223-induced effects towards cancer cells have been recently attributed to its N-tosyl group [11] and potentially involve hydrophobic interaction with DNA [12].

In the present study, we explored the mechanisms underlying LQB-223 antitumor effects in 2D and 3D models of breast cancer. We found a reduction in cell motility and in the migratory profile of MCF-7 and MDA-MB-231 cells following treatment with LQB-223, which was accompanied by an altered pattern of epithelial-mesenchymal markers. In 3D conformation, LQB-223 treatment remarkably decreased cell viability, as well as reduced tumor volume, and impaired cell migration. Notably, these effects were not seemingly observed following cell treatment with DOX, the prototype chemotherapeutic agent for breast cancer. LQB-223-mediated cytotoxic effects involved the modulation in the expression of survivin, XIAP, c-IAP1, and Mcl-1 anti-apoptotic proteins, molecules acting at the interface between chemoresistance and tumor progression. Conversely, survivin knockdown could potentiate the LQB-223 cytotoxic effects. Altogether, these results further indicate that LQB-223 impairs tumor growth and migration in 2D and 3D models of breast cancer, exhibiting different phenotypes and points, it as a promising compound against this neoplasia.

## 2. Results

### 2.1. Exposure to the LQB-223 Compound Modulates the Migratory Profile of Breast Cancer Cells

**Figure 1:**
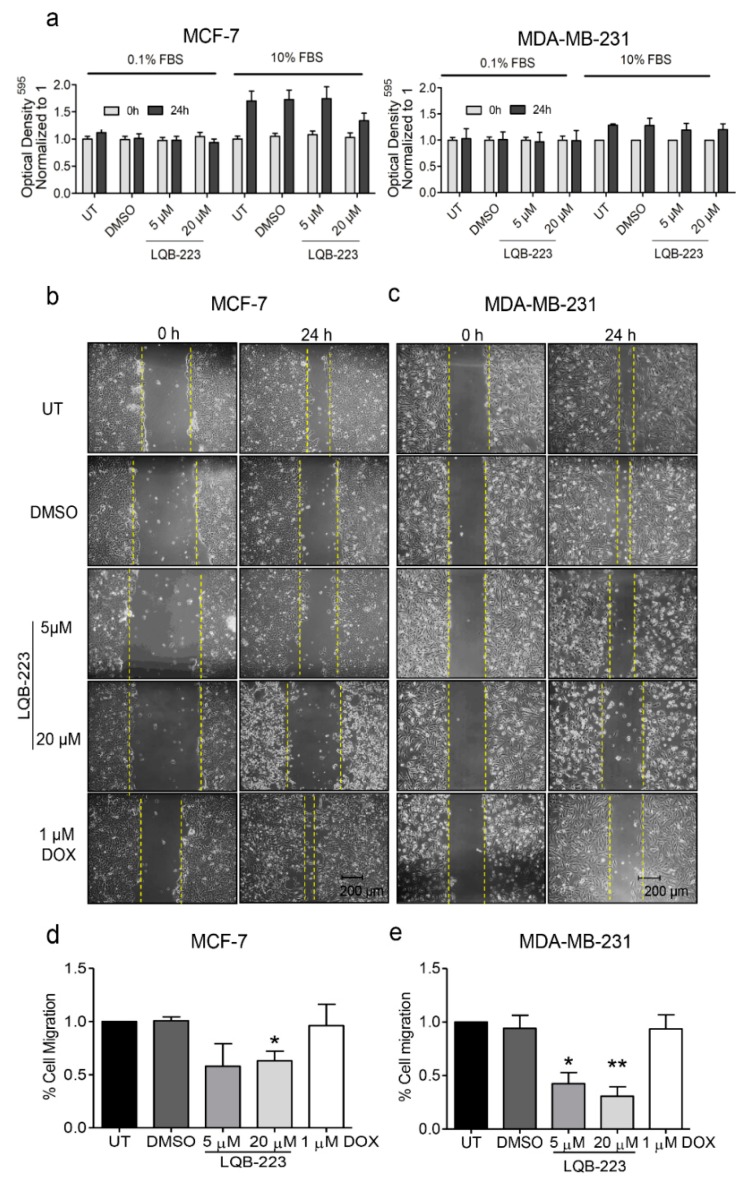
Assessment of proliferation in serum-deprived conditions and cell migration using the wound healing assay. (**a**) MCF-7 and MDA-MB-231 cells were cultured in DMEM containing 10% FBS or 0.1% FBS and treated with LQB-223 at the indicated concentrations for 24 h. Absorbance of cells stained with crystal violet was measured at 595 nm. (**b**) MCF-7 and (**c**) MDA-MB-231 cells were cultured in DMEM 0.1% FBS and treated with 5 or 20 µM of LQB-223 for 24 h. Wound closure was monitored and calculated using the software ImageJ. Yellow dotted line shows the gap area detected using ImageJ. Migration quantification of (**d**) MCF-7 and (**e**) MDA-MB-231 in percentage of covered area compared to the same region at 0 h timepoint. Values reported as mean and standard deviation of three independent experiments. Statistical significance was analyzed using the student’s t test (* *p* < 0.05; ** *p* < 0.01). UT: Untreated cells; DMSO: Dimethyl sulfoxide; DOX: Doxorubicin.

We have previously shown that the LQB-223 compound exhibited cytotoxicity to both non-invasive and invasive breast cancer cells [10]. This finding raised the question whether LQB-223 could modulate cell migration in addition to the induction of apoptosis and proliferation arrest. To address this issue, we cultured MCF-7 and MDA-MB-231 cells in serum-deprived conditions and performed the wound-healing assay following LQB-223 treatment. Our results show that directional cell migration was reduced upon exposure to 20 µM LQB-223 for both cell lines (Figure 1). For the metastatic MDA-MB-231 cell line, we found an inhibition in the migration profile even following treatment with 5 µM LQB-223, a concentration known to induce only late cytotoxic effects for this model. It is important to highlight that cell migration was assessed at 24 h of LQB-223 treatment, a time-point in which we observed no changes in crystal violet staining (Figure 1a). Remarkably, the inhibitory effects in the migration profile were not seemingly observed in DOX-treated cells, which retained the ability to move towards the wound area (Figure 1b–e). These data suggest that, differently from DOX, the LQB-223 compound more efficiently lowers breast cancer cell migration.

### 2.2. Cell Motility is Impaired in LQB-223-Treated Breast Cancer Cells

**Figure 2:**
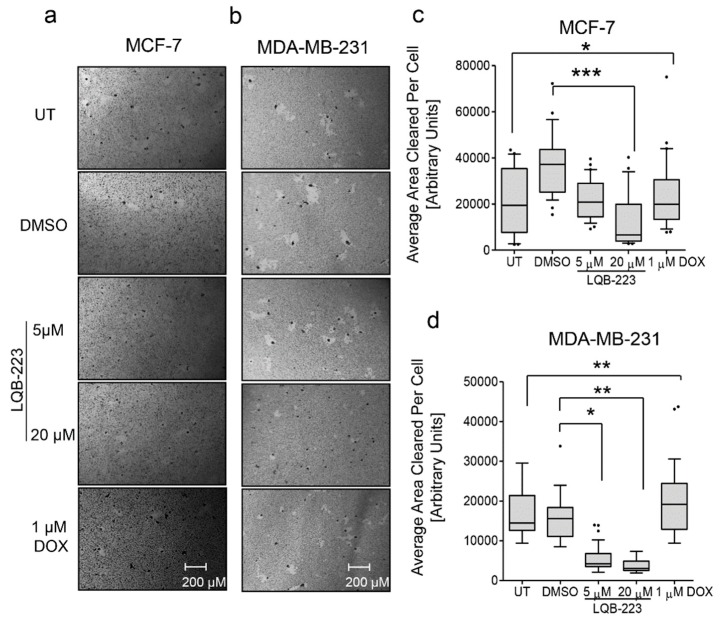
LQB-223 impairs motility of MCF-7 and MDA-MB-231 cells. (**a**) MCF-7 and (**b**) MDA-MB-231 cells were seeded onto 24-well plates coated with colloidal gold and treated with 5 or 20 µM of LQB-223 or 1 µM DOX for 24 h. The motility tracks were monitored under microscopy at 10× magnification and analyzed using the ImageJ software. Average area cleared per cell is shown for (**c**) MCF-7 and (**d**) MDA-MB-231 from three independent experiments. Statistical significance was analyzed using the one-way ANOVA test (* *p* < 0.05; ** *p* < 0.01; *** *p* < 0.001). UT: Untreated cells; DMSO: Dimethyl sulfoxide; DOX: Doxorubicin.

Next, we assessed whether LQB-223 could regulate cell motility, an essential feature of cancer cells, required as a first step in the movement from the primary organ to metastatic sites in distant organs [13]. For this purpose, cells at low-density were cultured in a gold colloidal surface and exposed to the LQB-223 compound. By measuring the area of phagokinetic track cleared by each single cell, chemokinesis (random motility) was quantitated. Figure 2 shows that LQB-223 exposure significantly reduced motility in both MCF-7 and MDA-MB-231 cells. Again, these effects were observed at lower concentrations for MDA-MB-231, suggesting that their motility abilities are more sensitive to LQB-223 treatment than MCF-7 cells. Notably, DOX treatment presented only slight effects on cell motility impairment (Figure 2), further confirming that DOX fails to prevent cell movement and migration of breast cancer cells. These findings suggest that LQB-223-mediated antitumor effects involve inhibition of the cell motility capacity of breast cancer.

### 2.3. Treatment with LQB-223 Inhibits Cell Viability and Growth of 3D Cell Models of Breast Cancer

**Figure 3:**
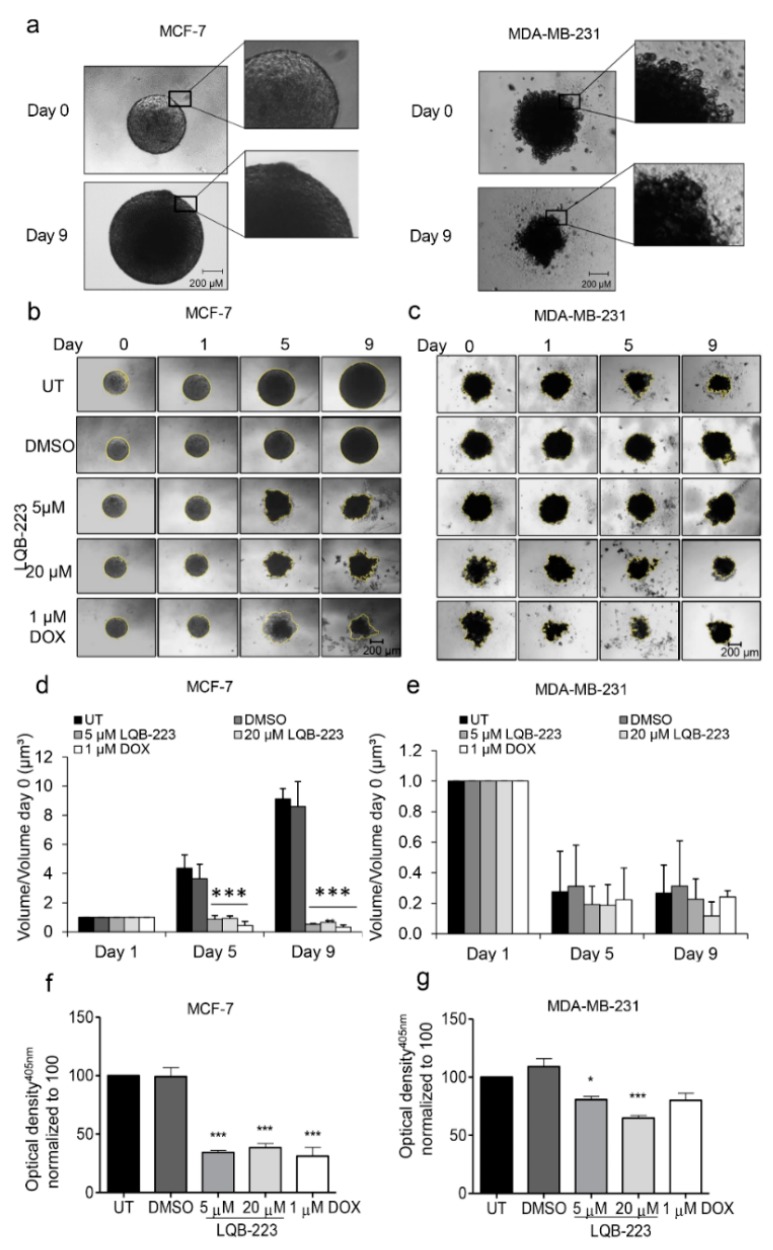
Cell viability and relative growth kinetics of 3D cultures after treatment with LQB-223 or DOX. (**a**) 3D structures of breast cancer cells were formed in non-adherent conditions. MCF-7 and MDA-MB-231 cells were seeded onto 96-well plates coated with 1.5% agarose and cultured for three days (day 0). The 3D cultures were cultivated for nine days and imaged at the timepoints indicated in each experiment (**b**) MCF-7 spheroids and (**c**) MDA-MB-231 cell aggregates were treated with LQB-223 or DOX and volume growth was analyzed on day 1, 5, and 9 after treatment. (**d**) Volume of MCF-7 spheroids and (**e**) MDA-MB-231 aggregates. The volume measurements were normalized to the volume of spheroids on day 0, and values are reported as mean and standard deviation of the fold change. Viability was assessed on day 9 using the APH assay. (**f**) Viability of MCF-7 spheroids after treatment with LQB-223 or DOX. (**g**) Viability of MDA-MB-231 aggregates after treatment with LQB-223 or DOX. Values reported as mean standard deviation of three independent experiments with at least eight replicates each. Statistical significance was analyzed using the Student’s *t* test (* *p* < 0.05; *** *p* < 0.001). UT: Untreated cells; DMSO: Dimethyl sulfoxide; DOX: Doxorubicin.

Our next step was to validate the findings concerning the cellular mechanisms induced by LQB-223 in tridimensional 3D culture models. Tridimensional models have been considered an important tool in drug discovery, displaying features of tumor growth in vivo in the early stage of development [14]. Beyond that, they better mimic physiological cell-cell interactions and resemble different phenotypes in a solid tumor due to the formation of an oxygen gradient [15]. Most importantly, 3D models were shown to be more resistant to drug treatment than monolayer culture, in which the cytotoxic effects of new drugs are generally overestimated [16]. Therefore, we initially set up experimental conditions for the formation of 3D structures using the liquid-overlay method. Formed tridimensional structures derived from MCF-7 and MDA-MB-231 cell lines showed morphological characteristics consistent with spheroids and compact aggregates (Figure 3a), respectively, according to a classification recently proposed by Froehlich and colleagues [17]. Following their formation, the 3D structures were exposed to LQB-223 treatment for nine days, when cell viability was measured. From the micrographs depicted in Figure 3b,c, we observed that the volume of untreated or DMSO-treated MCF-7 spheroids increased over the days, while LQB-223 prevented cell growth at both 5 and 20 µM doses. The same pattern was found for DOX-treated spheroids, which had their volume decreased over time, consistent with the well-established cytotoxic effect described by DOX in breast cancer cells. On the other hand, we observed that MDA-MB-231-derived compact aggregates exhibit a pattern of reduced volume over days in culture (Figure 3d,e). Nevertheless, the volumes of LQB-223-exposed structures were even smaller than the ones from non-exposed and DOX-treated (Figure 3d,e). Corroborating these data, the assessment of acid phosphatase activity revealed that 3D structures originated from both MCF-7 and MDA-MB-231 presented diminished viability when treated with the LQB-223 compound (Figure 3f,g). Besides that, MDA-MB-231 aggregates were less sensitive to DOX stimuli than MCF-7 spheroids. Altogether, these findings suggest that LQB-223 impairs growth and viability of tridimensional models of breast cancer.

### 2.4. Cell Migration is Prevented by LQB-223 Treatment of Tridimensional Cultures

**Figure 4:**
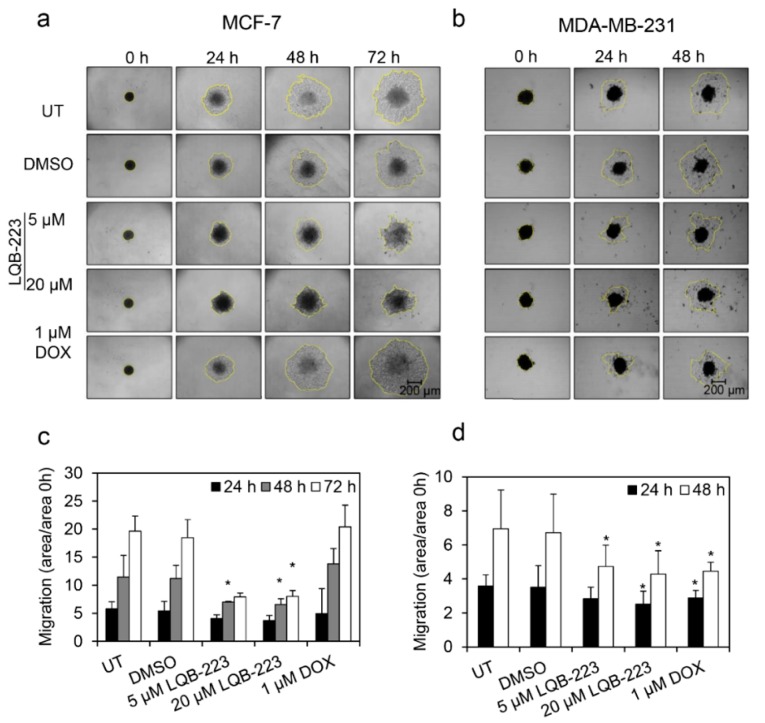
Analysis of cell migration cultivated as 3D cultures after treatment with LQB-223. (**a**) Fully-formed spheroids of MCF-7 or (**b**) aggregates of MDA-MB-231 were plated onto 24-well plates coated with gelatin, and migration was assessed over time upon exposure to LQB-223 or DOX. Area of cells migrated outwards from the 3D structure was calculated for (**c**) MCF-7 and (**d**) MDA-MB-231; aggregates were calculated and divided by the area of the 3D cultures on day 0. Values reported as mean and standard deviation of three independent experiments. Statistical significance was analyzed using the Student’s *t* test (* *p* < 0.05). UT: Untreated cells; DMSO: Dimethyl sulfoxide; DOX: Doxorubicin.

To reinforce the ability of LQB-223 to modulate the migratory capacity of breast cancer cells, we performed a gelatin-based migration assay in tridimensional structures. Figure 4 shows that MCF-7 and MDA-MB-231 cells migrated onto the gelatin surface over 72 h in 2% FBS conditions. Following treatment with LQB-223, MCF-7 cells fail to move outwards from the 3D structures, being confined to the center region where they were initially seeded. Interestingly, DOX-treated cells retain the capacity to migrate, in concordance with aforementioned results (Figure 4c,d). For MDA-MB-231 cells, LQB-223 exposure also results in decreased cell migration, but the differences between the effects induced by DOX are more discrete. These results suggest that cell migration is also inhibited in breast cancer 3D cultures.

### 2.5. LQB-223 Treatment Leads to Changes in the mRNA Levels of KRT18, CDH1, and C-MYC

**Figure 5:**
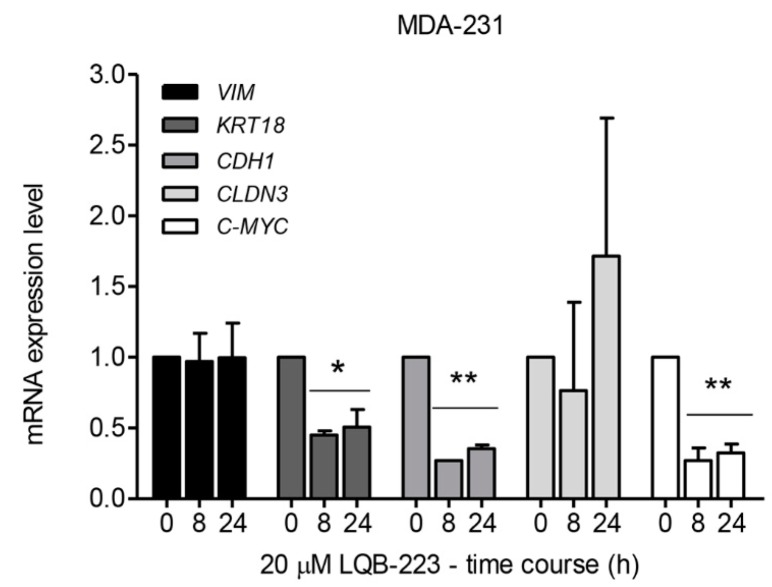
Effect of LQB-223 on epithelial-mesenchymal transition markers expression. MDA-231 cells were treated with 20 µM LQB-223 for 8 and 24 h. *VIM, KRT18, CDH1, CLDN3* and *C-MYC* mRNA levels were measured using qPCR and the 2^−ΔΔ*C*T^ method was used to calculate relative expression. The cells incubated with 20 µM LQB-223 for 8 and 24 h were compared with the cells without LQB-223 (0 h). *ACTB* was used as the endogenous control. Mean and standard deviation from three independent experiments are shown. One-way ANOVA followed by Bonferroni post-test (* *p* < 0.05; ** *p* < 0.01).

Since the LQB-223 compound was shown to impair migration in both 2D and 3D models of breast cancer, we next aimed to investigate whether it could modulate the epithelial-mesenchymal phenotype. To address this question, invasive and metastatic MDA-MB-231 cells were treated with LQB-223 for 8 and 24 h and the transcriptional levels of both epithelial and mesenchymal markers were analyzed. PCR analysis of transcripts encoding EMT markers revealed that LQB-223 exposure decreases, significantly, *CDH1* and *KRT-18* mRNA levels at 8 and 24 h and induces expression of *CLDN3* mRNA only at 24 h (Figure 5). On the other hand, *VIM* mRNA levels were not modulated with LQB-223 exposure for 8 and 24 h. Additionally, we observed a significant reduction of *C-MYC* mRNA levels (Figure 5), a regulator of the EMT phenotype. Altogether, these results implicate LQB-223 in the regulation of breast cancer cell plasticity.

### 2.6. LQB-223 Treatment Modulates Protein Expression of Apoptotic Regulators

**Figure 6:**
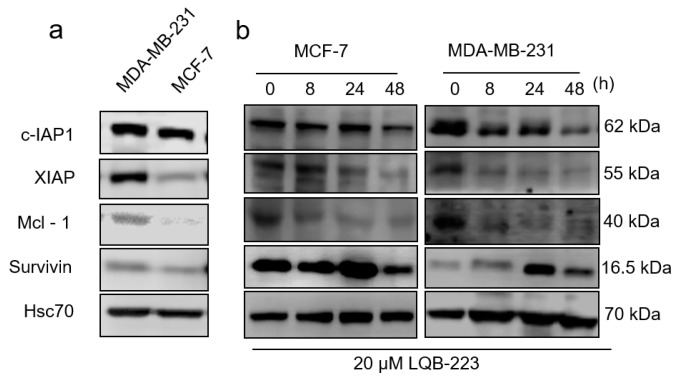
Modulation of chemoresistance-related proteins by the compound LQB-223. Protein levels were assessed by Western blotting assay of whole-cell lysates in MCF-7 and MDA-MB-231 cells. (**a**) Basal levels of apoptosis-associated proteins survivin, XIAP, cIAP-1, and Mcl-1 were compared between the MCF-7 and MDA-MB-231 cells. (**b**) MCF-7 or MDA-MB231 cells were treated with the LQB-223 compound for 8, 24, and 48 h and expression of c-IAP1, XIAP, Mcl-1, and survivin proteins was analyzed by Western blotting. Hsc70 was used as an internal control. Blots are representative of three independent experiments.

Next, we aimed to identify some of the molecular mechanisms underlying LQB-223-induced antitumor effects in breast cancer cells. For this purpose, we investigated proteins not only implicated in chemoresistance, but also acting at the interface between apoptosis evasion and tumor progression. Although classically described as inhibitors of apoptosis, cIAP-1, XIAP, Mcl-1, and survivin have been shown to modulate cell migration and metastasis in cancer models, including breast cancer [18,19]. Therefore, we initially assessed cIAP-1, XIAP, Mcl-1, and survivin basal protein content in the cell lines herein studied. These chemoresistance-related proteins were found more abundantly expressed in MDA-MB-231, compared to MCF-7 cells (Figure 6a). We then exposed both cell lines to LQB-223 treatment for a time-course of 8, 24, and 48 h. Our immunoblotting analysis showed that c-IAP1, XIAP, and Mcl-1 expression was consistently reduced upon LQB-223 exposure (Figure 6b). Conversely, survivin expression was initially upregulated at 8 and/or 24 h, but then had their levels decreased following prolonged treatment with LQB-223. Although with different kinetics, the pattern of protein regulation observed was quite similar between the cell lines (Figure 6b). These results suggest that the LQB-223 compound might modulate chemoresistance proteins while promoting its antitumor effects in breast cancer cells.

### 2.7. Survivin Inhibition Potentiates LQB-223-Induced Cytotoxicity in Breast Cancer Cells

**Figure 7:**
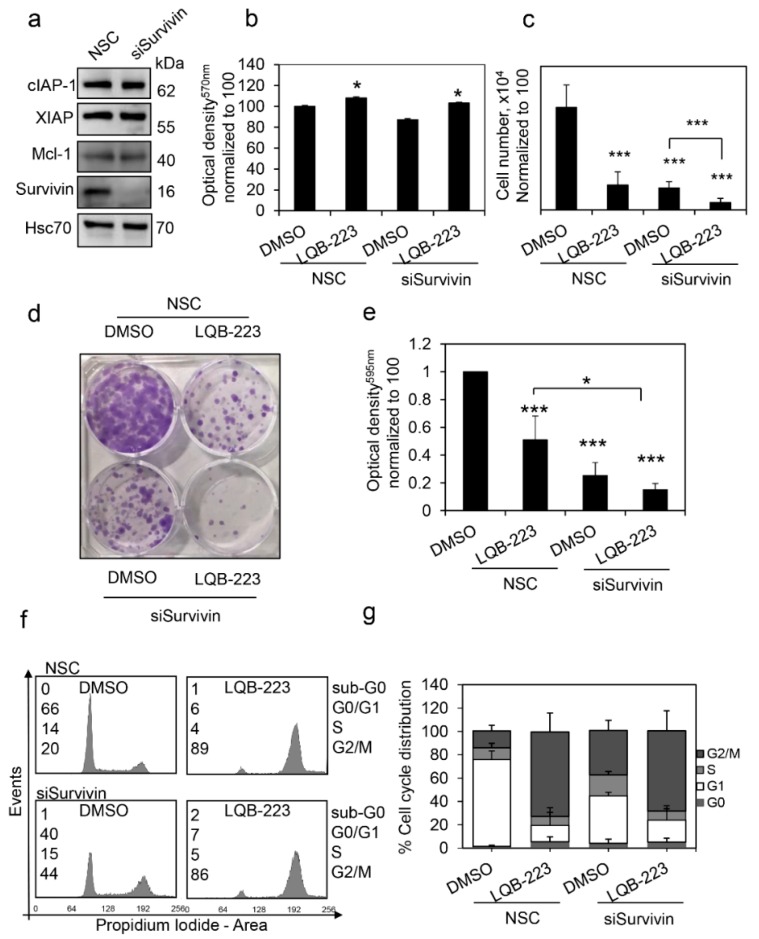
Analysis of the role of survivin in MDA-MB-231 cells’ sensitivity to LQB-223. (**a**) Modulation of chemoresistance-related proteins upon survivin inhibition in MDA-MB-231 Cells. (**b**) Cell viability of MDA-MB-231 cells after exposure to LQB-223 or/and survivin inhibition. (**c**) Cell count using trypan blue after exposure to LQB-223 or/and survivin inhibition. (**d**) Clonogenic assay after treatment with LQB-223 or/and survivin inhibition. (**e**) Absorbance of colonies of MDA-MB-231 stained with crystal violet shown in (**d**). (**f**) Analysis of cell cycle phases distribution after treatment with LQB-223 or/and survivin inhibition by means of flow cytometry. (**g**) Cell cycle distribution of MDA-MB-231 cells after treatment with LQB-223 or/and survivin inhibition. Values reported as percentage of cells in each cell cycle phase.. Values reported as mean and standard deviation of three independent experiments and statistical analysis were performed using the Student’s *t* test (* *p* < 0.05; *** *p* < 0.001).

Taking into consideration that survivin expression was induced following LQB-223 treatment, we questioned whether survivin could somehow interfere with LQB-223 sensitivity. To address this question, we silenced survivin expression with siRNA in MDA-MB-231 cells and compared them to cells transfected with a non-silencing control (NSC) with regards to response to LQB-223 exposure. Following survivin inhibition, we observed no modulation in the levels of XIAP, cIAP-1, and Mcl-1 antiapoptotic regulators (Figure 7a). Despite non-statistically significant differences in the MTT assay (Figure 7b), inhibition of survivin expression resulted in decreased cell number following LQB-223 exposure (Figure 7c). In addition, survivin-silenced cells exhibited discretely less clonogenicity in response to LQB-223 treatment than survivin-expressing cells (Figure 7d,e). As expected by its known function in cell cycle control, survivin inhibition increased the number of cells in the G2/M phase (Figure 7f,g). Interestingly, LQB-223-treated cells had their cycle arrested in G2/M, irrespective of survivin levels. Altogether, these results suggest that survivin overexpression can counteract LQB-223 cytotoxic effects, probably in a cell cycle-independent manner.

## 3. Discussion

Breast cancer is a heterogeneous type of neoplasm resulting in distinct phenotypical and morphological profiles that ultimately impact clinical outcome [2,3]. Here, we used cell lines with different metastatic potentials; the MDA-MB-231 line is a highly invasive and metastatic triple-negative model, whereas MCF-7 is a luminal non-invasive breast cancer cell line model [20,21]. The expression of estrogen receptor (ER), progesterone receptor (PR) and human epidermal receptor (HER) in breast tumors plays a role in crucial cellular events, like chemoresistance and invasiveness [22]. In addition, MCF-7 presents wild-type p53 protein, while MDA-MB-231 harbors mutant p53 with loss-of-function which confers poor prognosis and contributes to the MDR phenotype observed in some breast cancers [23].

Cell migration through the basal membrane is the first step in cancer metastasis, followed by colonization of distant organs [13,24]. Here, we report that treatment with LQB-223 decreased cell migration and motility of MDA-MB-231 and MCF-7 cells grown as monolayers. This effect was more pronounced in MDA-MB-231 than MCF-7 cells, suggesting that the genetic background of the latter confers a higher sensitivity to LQB-223 than the former. In silico studies suggested that the biological effects observed upon exposure to LQB-223 are explained by the n-tosyl moiety [11].

Several studies have shown that gene expression in 3D cell cultures better resemble clinical expression profiles than their 2D counterpart [25]. Like solid tumors in patients, their 3D architecture creates a gradient of nutrients and oxygen that ultimately affects expression of important genes associated with drug response, leading to an increase in resistance to some compounds [26,27]. Hence, 3D cell cultures can be used as a tool to test whether the biological effects observed in a 2D setting are maintained in a more complex 3D architecture [28]. Here, we show that 3D cultures from MCF-7 and MDA-MB-231 showed different basal degrees of tightness and morphology. MCF-7 formed tightly compacted spheroids, while MDA-MB-231 cells formed compact cell aggregates according to a classification proposed by Froehlich and colleagues [17]. The different morphology aspects observed in the cells grown as 3D culture could be explained by the expression of epithelial to mesenchymal transition (EMT) markers, as MCF-7 cells were more abundant for epithelial markers such as *CDH1*, *KRT18*, and *CLDN3*, whereas MDA-MB-231 cells expressed higher levels of mesenchymal markers, *CDH2*, *VIM*, and *SNAI1* [29,30,31]. A higher expression of epithelial markers increases cell-cell junctions and compactness. On the other hand, an increase of mesenchymal markers is associated with less compactness and less cohesive cell aggregates, as observed in MDA-MB-231 grown as spheroids [29,30]. In line with our findings using monolayer culture, we showed that LQB-223 had an antineoplastic activity towards breast cancer grown as 3D cultures, decreasing the volume, migration and cell viability of MCF-7 spheroids and MDA-MB-231 cell aggregates.

EMT promotes tumor progression, cell invasion, and metastasis. This process is typically defined by the upregulation of mesenchymal markers such as vimentin, claudin, and N-cadherin, and downregulation of epithelial markers like E-cadherin and cytokeratin-18 [31,32]. Since MDA-MB-231 cells is a highly invasive and metastatic triple-negative model, we set out to investigate if LQB-223 could revert to an epithelial phenotype via mesenchymal–epithelial transition (MET). Interestingly, despite the impairment of migration and motility, we observed a reduction of *CDH1* mRNA levels and no changes in expression levels of *VIM* after treatment with LQB-223. High levels of *VIM* can be associated with inhibition of *CDH1* expression, increasing cell migration and extravasation [33]. On the other hand, we observed that treatment with LQB-223 decreased *C-MYC* mRNA levels, corroborating previous results reported by our group that showed that LQB-223 impairs proliferation through arrest of the cell cycle in G2/M phase in MDA-MB-231 cells [10,34]. Of note, c-Myc plays an important role in several oncogenic events such as proliferation, regulation of cell cycle, angiogenesis, and EMT, inhibiting *CDH1* through miR-9 regulation [35]. In this context, the *C-MYC* modulation using LQB-223 could avoid a mesenchymal phenotype, inhibiting proliferation and migration in breast cancer.

Although some studies have shown that vimentin expression is a marker for poor prognosis [36], others have reported that its high expression can sensitize triple-negative breast cancer in vitro and in vivo to the c-Src inhibitor (dasatinib) [37,38]. Moreover, depletion of vimentin mitigates the c-Src inhibitory effects, suggesting that vimentin plays a role in the regulation of c-Src [38,39]. In line with our findings showing that LQB-223 reduced cell migration, treatment with LQB-223 reduced mRNA levels of *KRT18* and increased *CLDN3*. Cytokeratin-18 can be regulated by TGF-β1, acting as a tumor promoter; therefore, driving migration and metastasis, triggering snail and slug activation, and inducing EMT process [32,40]. Claudin-3 plays a role in tight junctions in the membrane surface between cells, and it was also detected intracellularly [41]. Low expression of claudin-3 is an important characteristic of the triple-negative breast cancer subclass. Evidence suggests that low expression of claudin-3 is associated with EMT induction [42,43], and claudin-low cell lines have higher expression of proliferation genes [44].

In contrast with the strong migration suppressive activity of LQB-223 towards breast cancer cells, treatment with DOX did not impair cell motility or migration of MCF-7 and MDA-MB-231 grown as monolayer or 3D cultures. DOX is an anthracycline used as a first-line treatment for breast cancer, inducing oxidative stress cytotoxicity and DNA damage through topoisomerase II inhibition [45,46]. Studies have shown that treatment with DOX at 4 µg/mL increased CXCR4 expression, a chemokine involved in metastasis in breast cancer [47]. Therefore, low concentrations of DOX in combination with another compound that reduces migration and motility could be a promising strategy to increase the success rate of breast cancer treatment. In combination with curcumin, low doses of DOX decreased mRNA levels of *CXCR4*, and combined with furanodiene, reduced cell migration and MMP-9-mediated invasion of MDA-MB-231 cells [47,48]. Jin and colleagues demonstrated that a combination of DOX and resveratrol inhibited cell growth and migration, reverting EMT properties of MCF-7/ADR cells, which provided a better outcome for DOX-resistant patients [49]. These observations indicate that the combination of DOX with new compounds is a promising strategy to improve cancer treatment through the bypassing of DOX-mediated effects in cell migration.

Recent data suggested an interplay between EMT and the MDR phenotype in breast cancer, showing a possible correlation between these two biological processes [50]. Here, we demonstrated that LQB-223 reduced cell migration and motility, and decreased Mcl-1, XIAP, and cIAP1 protein levels. Mcl-1 expression has been associated with chemoresistance and poor prognosis in breast cancer, and compounds that inhibit this protein can be a promising therapeutic strategy. For example, BH3 mimetics alone or combined with dasatinib inhibited invasion of MDA-MB-231 tumors [19]. Additionally, treatment with anacardic acid, a natural compound, decreased Mcl-1, vimentin, and MMP-9 protein levels and increased E-cadherin [51]. XIAP is another antiapoptotic protein and has been described as an important player in the invasion of lymph node and metastasis in patient samples [52]. Consistently, treatment with embelin inhibited XIAP and reduced the migratory potential of MCF-7 cells [53].

Interestingly, MCF-7 and MDA-MB-231 cells exposed to LQB-223 increased survivin protein levels at early time-points. Although involved in inhibition of apoptosis, survivin is an important regulator of cell cycle through interaction with chromosomal passenger proteins and stabilization of microtubules [54,55,56]. We suggest here that the increase in survivin protein levels could have a minor role in the molecular mechanisms involved in LQB-223-induced G2/M cell cycle arrest [10,57,58]. In order to investigate the role of survivin in cell response to LQB-223, we silenced survivin expression in MDA-MB-231 cells. survivin inhibition slightly decreased the ability of cells to form colonies, suggesting that the expression of this protein plays a role in long-term survival following treatment with LQB-223. Additionally, survivin inhibition led to a slight decrease of cell viability and cell count, suggesting that the therapy based on survivin knockdown could potentially enhance treatment effect. Many studies have shown that inhibition of survivin can sensitize cells to drug treatment. Downregulation of survivin enhanced paclitaxel-induced apoptosis in MCF-7 cells [56]. In MDA-MB-231 cells, survivin silencing by MX106/MX107 inhibited the oncogenic survivin function and decreased the cancer stem-like cell population, increasing drug sensitivity [59]. Altogether, these studies show that targeting survivin can improve drug response and, consequently, increase treatment success rates.

In conclusion, our results show that LQB-223 has a strong antineoplastic activity towards breast cancer cells, decreasing the metastatic potential of cell line models cultured as a monolayer or in 3D conformation. Since breast cancer is a heterogeneous disease, new therapeutic approaches are necessary to bypass the activation of intracellular processes that pose a threat to success of breast cancer chemotherapeutics. Through the downregulation of *C-MYC* and important anti-apoptotic proteins, LQB-223 emerges as a promising therapeutic strategy for breast cancer, inhibiting the growth and migration of tumor cells.

## 4. Materials and Methods 

### 4.1. Cell Culture

The MCF-7 (ATCC HTB-22) and MDA-MB-231 (ATCC HTB-26) adenocarcinoma cell lines were maintained in Dulbecco’s modified Eagle’s medium (DMEM-Gibco; Thermo Fisher Scientific Waltham, MA, USA) supplemented with 10% fetal bovine serum (FBS, Gibco; Thermo Fisher Scientific Waltham, MA, USA), penicilin and streptomicin (Gibco; Thermo Fisher Scientific Waltham, MA, USA) at a final concentration of 100 U/mL and kept in a humidified incubator at 37 °C and 5% CO_2_. Cells were routinely thawed from frozen stocks and subcultured for < 10 passages. Cell lines were periodically checked for mycoplasma contamination and cell authenticity was confirmed by genotyping of short tandem repeats.

### 4.2. Drug Treatment

The 11a-N-Tosyl-5-deoxi-pterocarpan LQB-223 compound was synthesized in the Laboratório de Química Bio-orgânica, at the Federal University of Rio de Janeiro, as previously described [9]. LQB-223 was dissolved in dimethyl sulfoxide (DMSO) at 25 mM and added to the cell culture medium at the final concentrations indicated in each experiment. The chemotherapeutic agent Doxorubicin (DOX; Rubidox R, Bergamo Ltda-Taboão da Serra, SP, BR) was kept at −20 °C and added to the cell culture medium at a final concentration of 1 µM for the indicated times in each experiment.

### 4.3. Wound Healing Assay

For assessment of drug treatment effect on cell migration, MCF-7 and MDA-MB-231 cell lines were plated at a final number of 3 × 10^5^ cells in full culture medium on 6-well plates for 24 h. The culture medium was then replaced by 0.1% FBS-containing medium in order to reduce cell proliferation by starvation. After 24 h in the presence of the starving medium, the surface of each well was scraped with a 10 µL sterile tip along the median axis at 3 different spots, allowing the measurement of 9 different migration points per experimental condition. Detached cells were removed by one PBS wash and cultures were treated with LQB-223 or DOX diluted in 0.1% FBS culture medium. The wounds were imaged using an inverted microscope (Axio Observer.Z1, Zeiss, Minneapolis, USA) and migration quantified using the software ImageJ [60] by measuring the wound area at 24 h after treatment/wound area at 0 h.

### 4.4. Crystal Violet Assay 

The cell lines were seeded at a final number of 10^4^ onto a 96-well plate and cultured in full culture medium containing 10% FBS or starvation medium containing 0.1% FBS. After cell adhesion (24 h), the control group was 10 min. The experimental groups were treated with LQB-223 or DOX for 24 h, fixed and equally stained with crystal violet. The dye was then dissolved in absolute methanol and absorbance was measured at 595 nm in the plate microplate reader (EZ Read 400 Microplate Reader, Biochrom, Holliston, MA, USA).

### 4.5. Phagokinetic Track Motility Assay

The drug effect in cell motility was assessed by tracking the ability of cells to clear gold from its path. For this, 24-well plates were coated with 300 µL of 1% BSA and incubated in a humidified CO_2_ incubator at 37 °C for 3 h followed by one wash with absolute ethanol. The wells were then coated with 300 µL of colloidal gold solution, prepared as follows: A total of 3.85 mL of sterile H_2_O, 630 uL of 14.5 mM AuHCl_4_, and 2.1 mL de 36.5 mM Na_2_CO_3_, followed by boiling at 100 °C for 5 min and addition of 0.1% of formaldehyde. The colloidal gold-coated plate was kept in the incubator for 24 h and cells were seeded at a final number of 2 × 10³ cells per well in full culture medium containing 10% until cell adhesion. Afterwards, the culture medium was changed to a starving medium containing 0.1% FBS and cells were treated with DOX or LQB-223 for 20 h. The wells were imaged using a microscope (Nikon Eclipse TS100, Nikon, Tokyo, Japan) coupled with a digital camera (Digital Sight DS-2 Mv, Nikon, Tokyo, Japan) and the tracks were quantified using the software ImageJ. A total of 20 tracks per experimental condition were randomly chosen for quantification.

### 4.6. Formation of 3D Cellular Structures 

For liquid-overlay spheroid formation, flat-bottom 96-well plates were coated with 1.5% agarose diluted in water and left at 4 °C for 15 min for agarose solidification. MDA-MB-231 and MCF-7 cells were plated at final number of 6000 and 2000 cells per well, respectively, in DMEM medium containing 10% FBS. Plates containing cells were then centrifuged at 400× *g* for 5 min and kept in a humidified incubator at 37 °C and 5% CO_2_ for 72 h. Full formation of 3D structures was confirmed microscopically and the subsequent experiments were initiated with a spheroid diameter between 300 and 500 µm.

### 4.7. Growth Kinetics and Viability of Tridimensional Structures

The growth of 3D cultures was assessed by quantification of volume in the course of 9 days starting from day 0, when the drugs were added to the 3D cultures. The acid phosphatase assay (APH) was the end-point analysis used to assess cell viability in untreated and treated samples, performed at day 9. After, fully-formed (day 0) 3D structures derived from MCF-7 and MDA-MB-231 cells were treated with the indicated concentrations of LQB-223 or DOX and imaged using the widefield microscope Nikon Eclipse TS100 coupled with a digital camera (Digital Sight DS-2 Mv, Nikon). Images were also taken at 24 h after treatment (day 1), at day 5, when the culture medium containing the specific drug concentration was changed for nutrient reposition and on the last day of the experiment (day 9). For relative growth quantification, the radius of each individual 3D structure was manually analyzed using the open-source ImageJ software. The volume was then calculated from the radius Volume=4/3×π×Radius³ and the relative growth was reported as Volume at treatment timepoint÷Volume at day 0.

For assessment of acid phosphatase activity, 3D structures were carefully transferred to an uncoated 96-well plate and cultured medium was removed by two PBS washes with PBS. The supernatant was then discarded and 100 µL of PBS was added to each well, followed by addition of 100 µL of APH assay buffer prepared prior to every experiment as follows: Nitrophenylphosphate (PNPP, 2 mg/mL, Sigma Aldrich, St. Louis, MO, USA), TritonX 0.1% vol/vol (Sigma Aldrich, St. Louis, MO, USA), and 0.1 M of sodium acetate; pH 4.8. Plates were kept at 37 °C for 90 min, protected from light, after which 10 µL of 1N NaOH were added to each well and absorbance was obtained at 405 nm using the plate reader (EZ Read 400 Microplate Reader-BioChrom, Holliston, MA, USA).

### 4.8. Gelatin-Based Migration Assay in 3D Conformation

To assess the effect of the LQB-223 compound and DOX in the migration of cells in 3D conformation, a gelatin-based assay. Briefly, 24-well plates were coated with 300 µL of 0.1% gelatin and left in a humified incubator at 37 °C for 45 min. The excess of gelatin was removed using a pipette. Fully-formed 3D structures were then transferred to the gelatin-coated plates followed by treatment with DMSO, LQB-223 or DOX. The migration of cells was assessed after 24, 48, and 72 h post treatment. In order to reduce proliferation that can confound the result analysis, the experiments were carried out in culture medium containing 2% FBS. Tridimensional structures were imaged using the widefield microscope Nikon Eclipse TS100 coupled with a digital camera (Digital Sight DS-2 Mv, Nikon). The migration index was quantified using the ImageJ software by measuring the area of cells migrating outwards the 3D structure divided by the corresponding area at 0 h.

### 4.9. Western Blotting

Protein extraction was performed using the Protein Extraction Buffer Invitrogen (Thermo Fischer Scientific, Waltham, MA, US). Twenty micrograms of protein were run on a 7%, 10%, or 12% SDS-PAGE gel, depending on the size of the protein, and transferred onto a nitrocellulose membrane (GE Healthcare) using a wet-blotting system (Bio Rad Laboratories) for 2 h at 100 V. Non-specific binding was blocked by incubating the membranes in 5% skimmed milk in TBS-Tween for 2 h. The blocked membranes were incubated overnight at 4 °C with the primary antibodies diluted in TBS/BSA 2% against FOXM1 (1:500, sc-502, Santa Cruz), XIAP (1:1000, 2045S, Cell Signaling), c-IAP 1 (1:2000, AF8181, R&D Systems), survivin (1:1000, 2808S, Cell Signaling), Mcl-1 (1:100, #4572, Cell Signaling) and Hsc70 (1:1000, sc-7298, Santa Cruz). After overnight probing with primary antibodies, the membranes were incubated with secondary antibodies anti-mouse (1:40000, GE Healthcare), anti-rabbit (1:40000, GE Healthcare), and anti-goat (1:2000, NB7362, Novus Technologies; Littleton, CO, USA) for 1 h at room temperature. Protein band signals were developed using the Clarity Max™ substrate (Western ECL Substrate-BioRad Laboratories, Hercules, CA, USA) and detected using the C-DiGit blot scanner (Li-cor Biociences, Lincoln, NE, USA).

### 4.10. Survivin Inhibition by Small Interfering RNA (siRNA)

For inhibition of survivin expression, MDA-MB-231 cells were transfected with 10 nM siRNA targeting survivin (siSurvivin) and a non-silencing control sequence (NSC) using lipofectamine RNAimax. After 24 h of transfection, cells were harvested, counted by trypan blue exclusion, and reseeded for LQB-223 treatment. Differences in cell viability between survivin-inhibited and control cells were compared by the MTT and clonogenic assays and flow cytometry DNA content analysis. Western blotting was performed for assessment of survivin levels.

### 4.11. Real-Time Quantitative PCR (qRT-PCR)

Total RNA from cell lines were extracted by Trizol reagent (TRIzol TM, Invitrogen, Carlsbad, CA, USA) according to the manufacturer’s instructions. RNA concentration and purity were measured using NanoDrop 1000 (Thermo Scientific, Waltham, MA, USA). Complementary DNA (cDNA) synthesis from 1 µg RNA was performed using the SuperScript^®^ II First-Strand (Invitrogen, Carlsbad, CA, USA). For real-time quantitative PCR (qRT-PCR), SYBR^®^ Green PCR Master Mix kit (Applied Biosystems; Thermo Fisher Scientific Waltham, MA, USA) was used according to the manufacturer’s instructions. The primer sequences utilized are described in Table 1, supporting information. The 2^−ΔΔ*C*T^ method was applied to quantify the relative expression levels between cells incubated with LQB-223 and controls, using ACTB as an endogenous reference. All PCR assays were done in duplicate using the 7500 Real-Time PCR System (Applied Biosystems; Thermo Fisher Scientific Waltham, MA, USA).

### 4.12. Statistical Analysis

Statistical analysis was performed using the GraphPad Prism software V5.0 (GraphPad Software, San Diego, CA, USA). The differences between the means of treatment-groups were tested using student’s *t* test or one-way ANOVA, followed by Bonferroni post-test. For median analysis, the Wilcoxon test was performed. A *p*-value < 0.05 was considered statistically significant and experiments were carried out in triplicates, unless otherwise stated.

## Figures and Tables

**Table 1:** Primer sequences used for qRT-PCR.

Gene	Protein	Forward 5’–3’	Reverse 5’–3’
*CDH1*	E-cadherin	GAATGACAACAAGCCCGAAT	GACCTCCATCACAGAGGTTCC
*CLDN3*	claudin-3	CTGCTCTGCTGCTCGTGTCC	TTAGACGTAGTCCTTGCGGTCGTAG
*VIM*	vimentin	GACAATGCGTCTCTGGCACGTCTT	TCCTCCGCCTCCTGCAGGTTCTT
*KRT18*	cytokeratin-18	GCGAGAAGGAGACCATGCA	GGTGTTCCCGGATTTTGATCT
*C-MYC*	c-Myc	AATGAAAAGGCCCCCAAGGTAGTTATCC	GTCGTTTCCGCAACAAGTCCTCTTC
*ACTB*	β-actin	GGCGGCACCACCATGTACCCT	AGGGGCCGGACTCGTCATACT
